# Improving women’s knowledge about prenatal screening in the era of non-invasive prenatal testing for Down syndrome – development and acceptability of a low literacy decision aid

**DOI:** 10.1186/s12884-018-2135-0

**Published:** 2018-12-17

**Authors:** Sian Karen Smith, Antonia Cai, Michelle Wong, Mariana S. Sousa, Michelle Peate, Alec Welsh, Bettina Meiser, Rajneesh Kaur, Jane Halliday, Sharon Lewis, Lyndal Trevena, Tatiane Yanes, Kristine Barlow-Stewart, Margot Barclay

**Affiliations:** 1 Psychosocial Research Group, Lowy Research Centre, C25, Prince of Wales Clinical School, Faculty of Medicine, UNSW Sydney, Corner High and Botany St, Kensington, Sydney New South Wales 2033 Australia; 2 Centre for Applied Nursing Research, School of Nursing and Midwifery, Western Sydney University, Ingham, Sydney, Australia; 3 South Western Sydney Local Health District, Institute for Applied Medical Research, Sydney, Australia; 4 Department of Obstetrics and Gynaecology, Royal Women’s Hospital, University of Melbourne, Parkville, Australia; 5 School of Women’s and Children’s Health, UNSW Sydney, Sydney, Australia; 6 Department of Maternal-Fetal Medicine, Royal Hospital for Women, Sydney, Australia; 7 Murdoch Children’s Research Institute, Royal Children’s Hospital, Parkville, Victoria Australia; 8 Department of Paediatrics, University of Melbourne, Parkville, Australia; 9 Sydney School of Public Health, The University of Sydney, Sydney, Australia; 10 School of Psychiatry, Faculty of Medicine, UNSW Sydney, Sydney, Australia; 11 Sydney Medical School, The University of Sydney, Sydney, Australia; 12 Women’s Services, Liverpool Hospital, Sydney, Australia; 13 Western Sydney University, Parramatta, Sydney, Australia

**Keywords:** Decision aid, Informed decision-making, Prenatal screening, Prenatal testing, Trisomy 21, Down syndrome, Non-invasive prenatal testing (NIPT), Low literacy

## Abstract

**Background:**

Access to information about prenatal screening is important particularly in light of new techniques such as non-invasive prenatal testing (NIPT). This study aimed to develop and examine the acceptability of a low literacy decision aid (DA) about Down syndrome screening among pregnant women with varying education levels and GPs.

**Methods:**

We developed a DA booklet providing information about first-trimester combined testing, maternal serum screening, and NIPT. GPs and women participated in a telephone interview to examine the acceptability of the DA and measure screening knowledge before and after reading the DA. The knowledge measure was designed to assess whether women had understood the gist of the information presented in the decision aid. It comprised conceptual questions (e.g. screening tells you the chance of having a baby with Down syndrome) and numeric questions (e.g. the accuracy of different screening tests).

**Results:**

Twenty-nine women and 18 GPs participated. Regardless of education level, most women found the booklet ‘very’ clearly presented (*n* = 22, 76%), and ‘very’ informative (*n* = 23, 80%). Overall, women’s conceptual and numeric knowledge improved after exposure to the DA, from 4% having adequate knowledge to 69%. Women’s knowledge of NIPT also improved after receiving the decision aid, irrespective of education. Most GPs found it ‘very’ clearly presented (*n* = 13, 72%), and that it would ‘very much’ facilitate decision-making (*n* = 16, 89%).

**Conclusions:**

The DA was found to be acceptable to women as well as GPs. A comprehensive evaluation of the efficacy of the decision aid compared to standard information is an important next step. Strategies are needed on how to implement the tool in practice.

**Electronic supplementary material:**

The online version of this article (10.1186/s12884-018-2135-0) contains supplementary material, which is available to authorized users.

## Introduction

Screening in early pregnancy for foetal abnormalities is an established part of routine care in Western countries [1]. Some countries, such as the UK and the Netherlands offer prenatal screening as part of their national screening programmes and costs are reimbursed [2, 3]. Other countries have not implemented screening programmes, but have policies in place to ensure women are made aware that screening tests are available [4]. Irrespective of a women’s age or family history, a nuchal translucency ultrasound, with or without a maternal serum test is generally available to women in early pregnancy to assess a woman’s risk of carrying a foetus with chromosomal abnormalities. The most common chromosome condition is Down syndrome which can lead to varying degrees of physical, behavioural, and cognitive problems. If an increased risk is identified, invasive diagnostic tests such as amniocentesis and chorionic villus sampling are available. Women could then be faced with the decision of terminating the pregnancy or preparing for the birth of a child with special needs [5, 6].

Following the discovery of cell-free foetal DNA in the mother’s blood, non-invasive prenatal testing (NIPT) is steadily becoming available to screen for Down syndrome in early pregnancy [7, 8]. NIPT is more accurate than conventional screening with a higher detection rate (99% versus ~ 80–90%, respectively) [9, 10], and eliminates the risk of miscarriage associated with invasive tests [11]. Accuracy, safety, and peace of mind are often cited as the perceived benefits of NIPT by women and health care professionals [12, 13]. NIPT has largely been driven by the commercial sector [14]. However, several European countries have evaluated the impact of integrating NIPT into existing prenatal screening programmes [15–17]. In Australia, the context for current study, women have to fund NIPT themselves as it is not available on Medicare or reimbursed through private insurance [18]. Existing Australian clinical guidelines state that all pregnant women should be offered NIPT as an option, and that health care professionals should be informed about NIPT and how it fits into current screening and diagnostic pathways [19]. As such, different approaches have been adopted in different practices, and information provision is likely to vary.

Access to decision support information in early pregnancy is essential for making informed decisions, particularly in light of the availability of NIPT and the perception that it is “just a blood test” [20, 21]. The hallmarks of such decisions are possessing good knowledge of the consequences of participating in, or declining screening, being able to deliberate the options and outcomes, and making a choice consistent with their attitudes [22].

Decision aids (DAs) are tools designed to help people to make informed health decisions by explicitly stating the decision, providing information about the potential benefits and risks, and by clarifying values [23]. In prenatal care, DAs increase knowledge, lower decisional conflict and reduce anxiety [24]. An environmental scan of publicly available prenatal testing DAs – a process whereby internal and external data sources were searched, namely the DA Library Inventory, correspondence with research networks, and electronic databases – found that none of the 20 DAs identified fulfilled the International Patient Decision Aid minimum standards [25]. Only a few DAs included values clarification exercises, and most were developed prior to the availability of NIPT, with the exception of a web-based multimedia decision aid [26]. In addition, prenatal screening DAs rarely acknowledge the involvement of health care professionals in decision-making. Yet, health care professionals play an important role in supporting informed reproductive choices. [27]. In Australia, General Practitioners (GPs) or primary care physicians, are well positioned to provide decision support information about prenatal screening as they are typically the first health professional that women meet in the early stages of pregnancy when screening decisions are made.

A key challenge when designing health information is making sure that it is accessible and understood to people with different education and literacy skills [28]. Even women with higher levels of education may not be familiar with the various screening tests and struggle to understand information if it not communicated clearly [29]. In Australia, low health literacy is common, and up to 60% of the general population experience difficulties accessing and understanding information [30]. While there are prenatal screening decision aids [26, 31], few have been specifically developed using low literacy design principles to help expectant couples better understand information on equitable basis regardless of their education and literacy level [32].

To address the gaps highlighted above, this descriptive study aimed to: (1) develop a low literacy DA about prenatal screening for Down syndrome including NIPT, to complement counselling by a GP or other antenatal health care professional, (2) examine the acceptability and comprehensibility of the DA among GPs and among women with different education levels, and elicit suggestions for improvement, and (3) measure women’s screening knowledge about the different screening tests available (including NIPT) before and after receiving the decision aid.

## Method

### Development of the low literacy DA

The DA was developed by a multidisciplinary steering committee (all authors) of health professionals and researchers with expertise in epidemiology, genetics education, genetic counselling, psychology, DA development, and maternal foetal medicine. Existing Australian prenatal screening resources and the International Patient Decision Aid Standards (IPDAS) informed the content [33–35]. The DA was designed as a written booklet because our previous work found that women preferred paper-based information [36]. It is intended to complement rather than replace information or counselling by a GP or other health care professional.

**Fig. 1:**
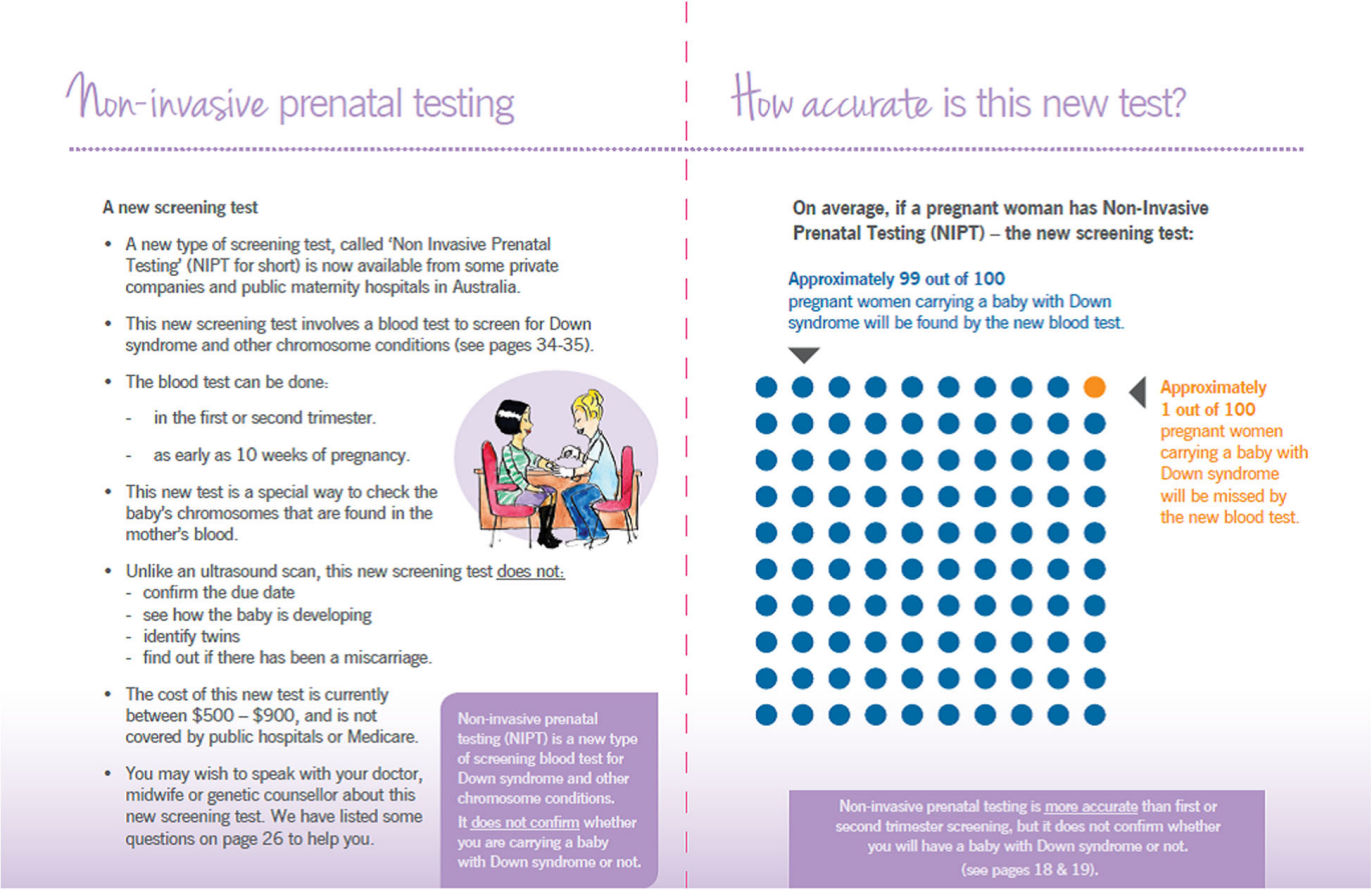
100-dot diagram illustrating the accuracy of NIPT

**Fig. 2:**
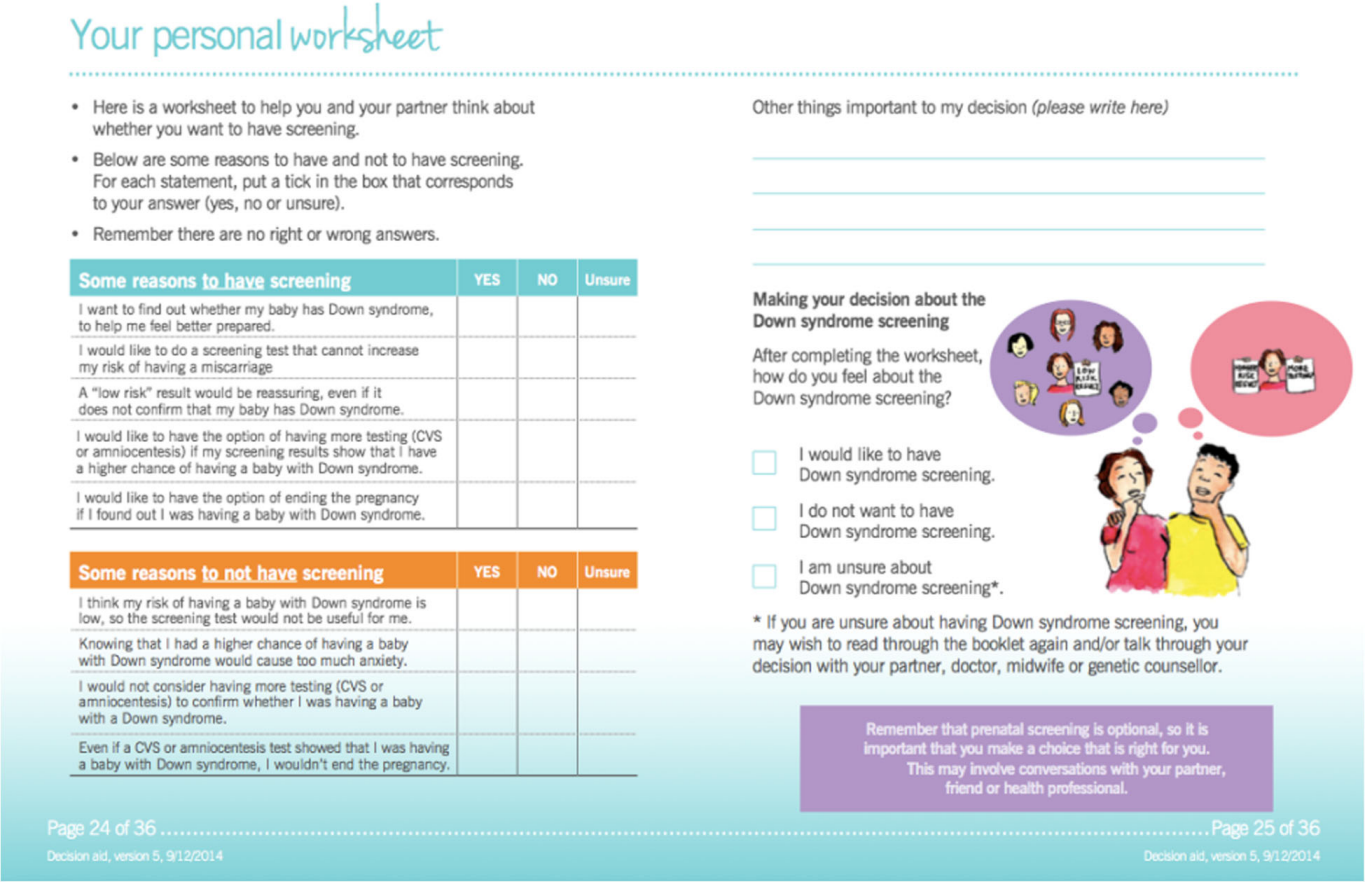
Personal worksheet included in the DA

Various low-literacy design strategies were applied, including; plain language; colour coding; glossary of medical terms; visual illustrations; simple medical diagrams; and providing contextual information before factual information [37–40]. The first draft of the DA was piloted with the target group, namely six pregnant women (mean age 31 years) with lower and higher education attending a hospital-based ultrasound clinic - 3 women with higher school certificate, and 3 had completed a university degree. The DA was then further refined based on their suggestions (e.g. using brighter colours and presenting culturally sensitive illustrations). Table 1 presents the DA content, including a 100-dot diagram (Fig. 1) and a worksheet (Fig. 2). The final version can be found – http://www.psychosocialresearchgroupunsw.org/decision-aids.html.

**Table 1:** Decision aid content

Component/feature	Description
General description	• Paper-based, 36-page, A5 booklet.
	• Written and quantitative information about prenatal screening (including NIPT) and diagnostic testing for Down syndrome.
Theoretical Framework	• Systemic functional linguistics were used to structure the information (Clerehan, Buchbinder et al. 2005).
Visual, format and design aspects	• Plain language, headings, bullet points, and white space were used.
	• Colour coding was consistently used to distinguish between screening and diagnostic tests.
	• A timeline was included to outline the timing of the different screening tests. Visual illustrations were used throughout the booklet to support key concepts and break up the text.
	• The image on the front cover was designed to set the scene for the booklet – a women contemplating the options.
	• Medical diagrams were presented of diagnostic testing.
Key factual content	• The difference between screening and diagnostic tests.
	• The chromosome conditions screened for.
	• The different types of screening tests (first-trimester, second-trimester and NIPT).
	• Accuracy of screening testing in detecting Down syndrome.
	• Diagnostic testing.
	• Options available after a diagnosis of Down syndrome.
	• Personal worksheet to help women/ couples decide about prenatal screening (Fig. 2).
Presentation of quantitative information	• Systematic 100-dot diagrams (see Fig. 1) used to convey sensitivity (rate at which women carrying babies with Down syndrome are detected) of the Combined First Trimester screening, Second Trimester Maternal Serum Screening and Non-Invasive Prenatal testing.

### Acceptability of the DA

#### GP recruitment and procedure

GPs were invited to provide feedback on the DA and assist in recruiting eligible women. Invitation letters were sent to 320 GPs working in antenatal shared care listed on a hospital database in South Western Sydney, New South Wales (NSW), Australia. South Western Sydney is a metropolitan part of Sydney, with a higher proportion of lower socioeconomic groups compared to other areas of Sydney [41]. In Australia, antenatal shared care is an arrangement where pregnancy care is shared between the GP and the hospital or other birth setting. GPs who offer shared care must have extra training and qualifications [42]. NSW does not have a state-wide prenatal screening programme and often women have to pay for screening tests privately. NSW Health policy states that women should be made aware of screening and be supported in making an informed decision [43].

Interested GPs returned an expression of interest form and were visited by researchers (AC and SS), who explained the recruitment process, obtained written informed consent, and provided information packs (recruitment sheet, participant information sheet and DA). GPs were asked to invite all eligible women, until the target sample size of 30 was reached. In our previous decision aid work, a sample size of 30 has been found to be suitable for obtaining feedback on the acceptability and comprehensibility of DAs [44, 45]. We also telephoned GPs individually about once a month to thank them for their commitment to the study, and to identify barriers to recruitment and discuss ways to boost recruitment.

GPs took part in a 30-min telephone interview using closed/structured and open-ended questions to obtain feedback on the DA. The current study reports the responses to the structured questions; the qualitative data will be reported in a separate paper. For participation, GPs received continuing professional development points from the Royal Australian College of General Practitioners.

#### Recruitment of pregnant women and procedure

Eligible women were invited by their GP and given an information sheet. Women were eligible if they were aged 16 years or older, were able to speak English and equal to or less than 13 weeks and 2 days of gestation. This cut-off time was chosen because in Australia combined first trimester screening is carried out between 9 weeks and 13 weeks (plus 6 days). This cut-off point enabled us to recruit women slightly later on in gestation to widen the recruitment window [46]. Women who expressed an interest were contacted by researchers (AC & TY) to determine eligibility, obtain verbal consent, and measure knowledge prior to receiving the DA. Eligible and consenting participants were mailed a copy of the DA and telephoned approximately 2 weeks later to collect socio-demographic information, elicit DA feedback, and measure knowledge. To increase recruitment, we offered women reimbursement for their time (a gift voucher), and sent a monthly newsletter to GPs to update and remind them to recruit potential participants.

Participants were recruited between 2014 and 2015. Ethics approval was obtained from the South Eastern and South Western Sydney Local Health District Human Research Ethics Committees (ref number: 12/219).

### Measures

#### GP measures

Socio-demographic data were collected from GPs. Closed questions (using Likert rating scales) adapted from our previous research were used to examine perceptions of its length, balance, clarity, and usefulness [47]. Some open-ended questions were asked to elicit feedback on how to improve the DA.

#### Women measures: Socio-demographic and obstetric variables

Data on age, marital status, educational level, country of birth, years lived in Australia, first language, employment status, occupation, previous pregnancy outcomes and screening experience were collected.

#### DA acceptability and comprehensibility

Closed questions using Likert rating scales examined the acceptability of the DA in terms of its length, balance, clarity, and amount of information. Comments on the illustrations, colour coding, diagrams, and worksheet were elicited using open-ended questions, and participants were asked to offer suggestions on how to improve the DA.

#### Knowledge

Knowledge is a key component of informed decision-making. Prenatal screening knowledge was measured to identify the extent to which women’s knowledge changed before and after exposure to the DA, and examine whether the resource would help inform women’s decision-making during pregnancy. The knowledge questions and marking scheme were informed by Fuzzy Trace Theory, a dual-process theory which proposes two ways in which people process and store information in their memory: (i) processing and remembering the bottom-line meaning (‘gist’), and (ii) processing and recalling information precise details (‘verbatim’) [48]. When making decisions, studies have shown that gist information processing tends to be superior to verbatim processing in improving the quality of the decision, possibly because it relies less on remembering the exact details [49] As such, our knowledge measure was designed to assess whether women understood the ‘gist’ of the information, including their (i) conceptual knowledge of screening for Down syndrome (e.g. screening tells you the chance of having a baby with Down syndrome) and, (ii) numeric knowledge (e.g. the accuracy of different screening tests). Nineteen items with true/false response options, adapted from previous questions developed by [50] and our previous work [51]. Existing knowledge measures did not include questions about NIPT so we developed our own items. A scoring scheme was developed providing a maximum score of 22 (16 for conceptual knowledge and 6 for numeric knowledge) (Additional file 1 knowledge scoring scheme and questions). It was decided a priori that a score of 75% or above (score ≥ 17 out of 22) would be considered ‘adequate knowledge’. Although the 75% knowledge threshold might be considered high, it enabled us to identify whether the conceptual and numeric knowledge scales were responsive to exposure to the DA. Thus, to achieve ‘adequate knowledge’, participants needed to answer a combination of conceptual and numeric questions correctly.

### Data analysis

Data were analysed using SPSS 24.0 (Statistical Program for the Social Sciences. Basic descriptive statistics, including means, medians, percentages, ranges and standard deviations were calculated to describe the samples in terms of socio-demographic characteristics.

## Results

### GP sample characteristics

Twenty-two GPs returned an expression of interest form and 18 GPs consented. The mean age of the GPs was 45 years (range 28–66 years), 72% were female, 50% were born in Australia, and 40% had more than 20 years’ experience (Table 2).

**Table 2:** GP Characteristics (*n* = 18)^a^

Characteristics	*N* (%)
Age (years)	
Mean	45
Range	28–66
Gender	
Male	5 (28)
Female	13 (72)
Country of birth	
Australia	9 (50)
South-East Asia	5 (33)
Africa	3 (16)
UK	1 (6)
Primary language	
English	11 (61)
Other	7 (39)
Years of experience as a GP	
Less than a year	3 (20)
1–5 years	1 (7)
6–10 years	2 (13)
11–20 years	3 (20)
21–30 years	5 (33)
31+ years	1 (7)
Employment status	
Full-time	13 (72)
Part-time	5 (28)
Current role in general practice	
Registrar/in training	4 (22)
Contractor/sessional	7 (39)
Retainer/salaried	3 (17)
Partner/principal	4 (22)

^a^Some percentages do not add up to 100% due to rounding

### Women sample characteristics

All of the GPs managed to invite at least one woman to participate. A total of 59 pregnant women were initially invited to take part. Of these, 22 did not meet the eligibility criteria and 8 were not interested. A total of 29 eligible women agreed to participate.

The mean age of the women was 32 years (range 25–40 years) (Table 3). Seventeen of the 29 women (59%) had a university education, 7 (24%) had a trade or technical qualification, and 5 (17%) had completed high (secondary) school education (aged between 15 and 16 years) or had achieved a Higher School Certificate. The Higher School Certificate (HSC) is the qualification awarded to senior high school students in Years 11 and 12 (aged between 16 to 18 years) who successfully complete high school. Participants were divided into two groups – (i) Higher education (*n* = 17, 59%) – undergraduate or postgraduate degree and, (ii) Lower education (*n* = 12, 41%) – completed secondary/ high school education or had successfully completed a Higher School Certificate, or trade/technical/vocational qualifications.

**Table 3:** Characteristics of the women (*n* = 29)^a^

Characteristics	*N* (%)
Socio-demographic characteristics	
Age (years)	
Mean	32
Range	25–40
Marital Status	
Married	24 (83)
Living with partner	4 (14)
Not living with partner	1 (3)
Educational level	
Year 10 or below^b^	3 (10)
Year 12 Higher School Certificate (HSC)^c^	2 (7)
Trade or technical certificate	7 (24)
Bachelors/Undergraduate degree	10 (35)
Postgraduate degree	7 (24)
Country of birth	
Australia	22 (76)
Other	7 (24)
Language/s spoken at home	
English only	19 (66)
Bilingual	10 (34)
Current employment status	
Full-time employed	14 (48)
Part-time employed	7 (24)
Self-employed	3 (10)
Homemaker	4 (14)
Student	1 (3)
Has private health insurance	
Yes	22 (76)
No	6 (21)
Obstetric variables	
First pregnancy	
Yes	13 (45)
No	16 (55)
Previous pregnancy outcomes	
Previously experienced a miscarriage	7 (24)
Previously experienced a termination of pregnancy	2 (7)
Previous screening tests	
Yes	9 (31)
No	5 (17)
Unsure	3 (10)
Previous diagnostic tests	
No	16 (55)
Unsure	1 (3)

^a^Some percentages do not add up to 100% due to rounding

^b^In Australia, Year 10 is the 10th full year of compulsory secondary (or high school) education with students aged between 15 and 16 years. By the end of Year 10, all qualifying students complete secondary (high) school

^c^Senior secondary (high) school education runs from Year 11 and Year 12 with students aged between 16 to 18 years. The Higher School Certificate (HSC) is the qualification awarded to senior secondary school students who successfully complete senior high school

### Acceptability of the DA

#### GPs

Most GPs felt that the information was ‘very’ clear (*n* = 13, 72%), ‘very’ easy to read (*n* = 13, 72%), ‘very’ useful (*n* = 16, 89%), and ‘very’ appealing (*n* = 14, 78%) (Table 4). All GPs described the DA as ‘very’ informative (*n* = 18, 100%). The majority of GPs felt that the DA presented a balanced view of prenatal screening (*n* = 16, 89%), and would ‘very much’ assist women/couples in helping them to better understand about screening (*n* = 15, 83%) and facilitate their decision-making (*n* = 16, 89%).

**Table 4:** GP responses regarding the acceptability of the DA (*n* = 18)

Questions and responses options	*N* (%)
How clear was the information?	
Very	13 (72)
Somewhat	5 (28)
How informative was the DA?	
Very	18 (100)
How easy to read was the DA	
Very	13 (72)
Somewhat	3 (17)
Not at all	2 (11)
How useful was the DA?	
Very	16 (89)
Somewhat	2 (11)
How appealing was the DA?	
Very	14 (78)
Somewhat	4 (22)
How would you describe the amount of information?	
Just right	9 (50)
Too much	9 (50)
How balanced did you find the information?	
Completely balanced	16 (89)
Encouraging prenatal screening	2 (11)
Will the DA make it easier for you to communicate with patients?	
Very much	8 (44)
Somewhat	8 (44)
Not at all	2 (11)
Do you think the DA will assist women/couples in helping them to understand about prenatal screening?	
Very much	15 (83)
Somewhat	3 (17)
Do you think the DA will assist women/couples in making decisions?	
Very much	16 (89)
Somewhat	2 (11)
How feasible would it be to implement the DA into routine practice?	
Very	10 (56)
Somewhat	5 (28)
Not very	3 (16)

Most felt the DA would make it ‘somewhat’ or ‘much’ easier to communicate to women about screening (*n* = 16, 88%). Just over half of GPs (*n* = 10, 56%) thought it would be ‘very’ feasible to implement the DA into practice, whereas the remaining GPs felt it would be ‘somewhat’ more challenging (*n* = 8, 44%). Half of the GPs described the information in the DA as just the right amount (*n* = 9, 50%), and the other half thought it would be ‘too much’ for women (*n* = 9, 50%).

#### Women

Overall, women reacted positively towards the DA (Table 5). The majority of participants reported reading all the information (*n* = 25, 86%), and that it took them less than 30 min to read (*n* = 23, 79%). Around half of the women reported that ‘all’ or ‘most’ of the information was new to them (*n* = 15, 51%). Most women felt the DA presented a balanced view of screening (*n* = 21, 72%), although a slightly higher proportion of women with higher education thought it was encouraging screening (*n* = 6, 35%) compared to those with lower education (*n* = 2, 17%). Most women thought the amount of information was ‘just right’ (*n* = 20, 71%), with only a few finding it to be ‘too much’ information (*n* = 3, 11%).

**Table 5:** Women’s responses regarding the acceptability of the decision aid by higher and lower education groups^a^

Questions and response options	Higher education (*n* = 17)	Lower education (*n* = 12)	Total women (*n* = 29)
	*N* (%)	*N* (%)	*N* (%)
How much of the DA did you read?			
I read all of it	14 (82)	11 (92)	25 (86)
I read part of it	0 (0)	1 (8)	1 (3)
My GP went through it with me	1 (6)	1 (8)	2 (7)
How long did it take you to read it?			
Less than thirty minutes	14 (82)	9 (75)	23 (79)
More than thirty minutes	3 (18)	3 (25)	6 (21)
What about the amount of information?			
Not enough	3 (18)	2 (18)	5 (18)
Just right	12 (71)	8 (73)	20 (71)
Too much	2 (12)	1 (9)	3 (11)
How balanced was the information?			
Encouraging prenatal screening	6 (35)	2 (17)	8 (28)
Completely balanced	11 (65)	10 (83)	21 (72)
Order of topics presented			
I liked the order	13 (89)	11 (92)	24 (89)
I’m not sure	2 (13)	1 (8)	3 (11)
How much of the information was new?			
All or most	10 (59)	5 (41)	15 (51)
Some	7 (41)	6 (50)	13 (45)
None	0 (100)	1 (8)	1 (3)
Did you show the booklet to anyone?			
Yes	11 (65)	7 (58)	18 (62)
No	6 (35)	5 (42)	11 (38)
Who did you show the booklet to?			
Husband/partner	9 (53)	6 (50)	15 (52)
Friend who is pregnant	0 (0)	1 (8)	1 (3)
GP + husband/partner	2 (12)	0 (0)	1 (7)
Would you recommend the DA?			
Yes I would	14 (82)	9 (75)	23 (79)
I’m not sure	2 (12)	2 (17)	4 (14)
No	1 (6)	1 (8)	2 (7)
How worried you felt after reading the DA?			
Not at all	8 (47)	7 (58)	15 (52)
A little bit	5 (29)	5 (42)	10 (35)
Somewhat	4 (24)	0 (0)	4 (14)
Did you use the worksheet?			
Yes	5 (29)	5 (42)	10 (34)
No	12 (71)	7 (58)	19 (66)
Please indicate if you thought the booklet was…			
Clearly presented	12 (71)	10 (83)	22 (76)
Informative	13 (77)	10 (83)	23 (80)
Easy to read	13 (77)	11 (92)	24 (83)
Useful	14 (82)	10 (83)	24 (83)
Appealing to look at	10 (59)	9 (75)	19 (66)
How helpful was the DA in terms of…			
Increasing understanding of options	15 (88)	10 (83)	25 (86)
Clarifying the benefits of each option	9 (53)	9 (75)	18 (62)
Clarifying the risks of each option	12 (71)	8 (67)	20 (69)
Clarifying your decision-making	8 (47)	8 (67)	16 (55)
Helping you reach a decision	8 (47)	6 (50)	14 (48)

^a^Some percentages do not add up to 100% due to rounding

Most women found the booklet ‘very’ clearly presented (*n* = 22, 76%), ‘very’ informative (n = 23, 80%), ‘very’ easy to read (*n* = 24, 83%), and ‘very’ useful (n = 24, 83%) (Table 5). Slightly fewer women described the DA as ‘very visually appealing to look at’ (*n* = 19, 66%). The majority of women felt the booklet was ‘very’ helpful in increasing their understanding of the options (n = 25, 86%). Around half of the women reported it being ‘very’ helpful in clarifying their decision-making (*n* = 16, 55%) and reaching a decision (*n* = 14, 48%). Just over a third of women reported completing the personal worksheet (*n* = 10, 34%).

#### Women’s responses to the open-ended questions

All of the women found the font size appropriate. The colours were described as ‘bright’, and ‘consistent’. The illustrations and diagrams were generally well received; being described as ‘appropriate’, and ‘a good mix of cultures’. However, a few women did not like the illustrations and found them ‘condescending’ and too ‘upbeat’ considering the serious nature of the topic.

The summary sheet, timeline, worksheet and glossary of medical words were described as ‘useful’, and helping to ‘put it into perspective’. The 100 dot-diagrams (Fig. 1) received mixed responses – some described them as ‘a clever visual representation’, whereas others thought they were ‘difficult to grasp’ in terms of what they were representing (i.e. pregnant women carrying a baby with Down syndrome).

#### Women’s screening knowledge – Conceptual and numeric

Overall, there was a difference in women’s knowledge before and after exposure to the DA, with mean scores increasing from 12.7 (out of 22) to 18.3 (Table 6). Conceptual knowledge scores improved from 12.0 to 14.4 (out of 16), and numeric knowledge scores increased from 0.7 to 3.9 (out of 6). Women’s knowledge about NIPT also improved from 2.1 to 4.1 (out of 5) after receiving the decision aid. Both education groups showed improvements, and a slightly higher proportion of women with higher education had increased knowledge compared to women with lower education (77% versus 58%, respectively).

**Table 6:** Women’s conceptual and numeric screening knowledge before and after receiving the decision aid by education group

	Higher education (*n* = 17)	Lower education (*n* = 12)	Total women (*n* = 29)
Pre Knowledge Scores, Mean (SD)^a^			
Conceptual (maximum score 16)	12.1 (1.7)	11.9 (2.2)	12 (1.9)
Numeric (maximum score 6)	0.9 (1.4)	0.3 (0.7)	0.7 (1.2)
NIPT knowledge (maximum score 5)	2.1 (1.2)	2.2 (0.6)	2.1 (1.0)
Total knowledge score (max score n)	13.1 (2.3)	12. 2 (1.8)	12.7 (2.1)
Adequate knowledge^b^ (total score ≥ 17 out of 22), *n* (%)	1(6)	0 (0)	1(4)
Post Knowledge Scores, Mean (SD)			
Conceptual (maximum score 16)	14.7 (1.4)	14.1 (1.5)	14.4 (1.4)
Numeric (maximum score 6)	4.2 (2.4)	3.4 (2.4)	3.9 (2.4)
NIPT knowledge (maximum score 5)	4.3 (1.0)	3.8 (1.1)	4.1 (1.09)
Total knowledge score (max score 22)	18.9 (3.2)	17.5 (3.4)	18.3 (3.3)
Adequate knowledge (total score ≥ 17 out of 22), *n* (%)	13 (77)	7 (58)	20 (69)

^a^Data missing for 1 participant

^b^Participants were classified as having ‘adequate’ or ‘inadequate’ knowledge using the midpoint of the scale. It was decided a priori that a pass mark of 75% or above (score ≥17 out of 22) would be considered ‘adequate knowledge’

#### Key revisions to the DA based on participant feedback

Both women and GPs had useful suggestions on how to improve the booklet. The following revisions were made in accordance with their feedback. We included a summary page comparing the accuracy and potential risks (i.e. miscarriage) of the different types of screening and diagnostic tests so the reader had all the information in one to which they could refer to in their decision-making. Both women and GPs wanted more practical information on where NIPT could be done and who to ask about it; we included a list of questions about NIPT that women could ask their GP, or other antenatal health professional. Some women were interested to know where they could find more information on the practicalities or impact of raising a child with Down syndrome. As such, in our revisions, we provided details of several resources produced by Down syndrome Australia. Some GPs felt the booklet should explain that screening requires medical referral, and clarify that screening identifies other chromosomal conditions. Throughout the booklet, we made some wording changes in accordance with Down syndrome Australia protocol on how to communicate with the public about Down syndrome. For example, we replaced the term ‘risk’ with ‘chance’, and ‘problem’ or ‘disease’ with ‘condition’. Based on GP feedback, we described diagnostic tests as ‘definite’ tests and replaced ‘detecting Down syndrome’ with ‘identifying Down syndrome’. We also reworded the 100-dot diagrams to clarify that the diagrams represented 100 pregnant women carrying a baby with Down syndrome.

### Discussion

This article describes the development and acceptability of a low literacy DA about Down syndrome screening. The DA was well received by women with different education levels and GPs. Women’s knowledge of the different types of screening tests, including NIPT, increased after exposure to the DA. Most women felt the information was very clearly presented, easy to read, informative, and rated the amount of information and length favourably. The DA was considered to be relevant, with most reporting they would recommend it to others. Similarly, most GPs reported the DA as clear, and that it would assist women in decision-making.

Although conceptual and numeric knowledge increased for both higher and lower education groups, a slightly higher proportion of women with higher education were found to have adequate knowledge compared to those with lower education. Although this is a small sample, it nonetheless echoes previous work showing that women from lower education groups experience greater difficulties making an informed decision [32]. Similarly, women with higher education have shown to benefit more from decision aids than those with lower education, possibly because they are more familiar with the engaging in decision-making and critically appraising health information [33, 47, 52].

Both women and GPs reviewed the DA positively, and there were few discrepancies in their views towards it. However, they did differ with regard to the amount of information, with half of the GPs thinking there was too much information, yet most women thinking it was just about right. Women expressed the need for more experiential information about living with a child who has Down syndrome. The final version includes website links with information about this. It is not uncommon for health professionals to underestimate how much information patients want to receive, possibly through fear of overloading patients, or underestimating their understanding [53].

Not all GPs agreed that the DA would be easy to implement and some felt it would not necessarily facilitate communication. The challenges of implementing DAs in clinical practice are widely reported [54–56]. Although DAs have proven to be effective, they are not commonly used in practice because of communication, cultural, ideological, organisational, and practical barriers [57]. Lepine et al. (2016) identified a number of factors influencing whether health professionals would use a prenatal screening DA, ranging from whether the tool was positively appraised, considered relevant, or being readily accessible to having enough time and colleagues endorsing the tool [58].

Our results showed that around two-thirds of women reported not using the values clarification worksheet. Perhaps the information on its own without a values clarification exercise might be enough to improve knowledge and enhance decision-making [27, 59, 60], or women found it difficult to complete. There is debate about whether DAs interfere with intuitive forms of information processing, and exercises that encourage deliberative (slow and analytic) decision-making may not always lead to better decisions. Clearly, more research is needed as to explore the factors that influence people using values clarification exercises, who might benefit more from such methods, and whether information alone might be enough to clarify values.

We note that GPs were the only health professionals involved in the study. Including other health professionals (e.g. midwives, obstetricians, and genetic counsellors) would have been useful to explore the diversity of perspectives. One study observed that health professionals varied in their attitudes towards using prenatal screening DAs; midwives seemed more positive about using DAs compared to GPs and obstetricians [58].

At the time of the study, NIPT was on the cusp of being implemented into the Australian healthcare system, predominately in the private health care system with test results being sent to offshore laboratories on a user pays basis with no reimbursement. At present, NIPT is more widely available in both the private and public healthcare system, and although it is less expensive than it used to be, it is still offered with no reimbursement. It is possible that discussions with health care professionals about NIPT may have influenced women’s knowledge and understanding about NIPT, and women may have paid less attention to the decision aid because NIPT was too expensive for women to consider a viable option. However, we note that some GPs in our study were not fully aware of the availability of NIPT and found the decision aid to be informative in improving their own knowledge. Further, our results showed that women’s knowledge of NIPT increased after exposure to the decision aid and they were able to correctly answer questions about accuracy of the NIPT that would have required reading the decision aid.

This study has a few limitations. Firstly, despite efforts to recruit partners to the study we were not successful. However, most women in the current study reported showing the DA to their partners, indicating that they valued their involvement. Similarly, previous research has shown screening decisions are often made by couples together [27, 36, 61, 62]. Others have also highlighted the challenges of recruiting men to participate in health research. Strategies have been proposed to help overcome men’s resistance to participation, these include; emphasising the personal benefits and altruistic elements of the research, simplifying technical information, and using humour [63]. Future research should consider these recruitment strategies to ensure partners’ perspectives are taken into account.

Secondly, although we made efforts to recruit women with varying education levels to ensure the tool was acceptable to different education groups, we encountered difficulties recruiting women from lower education groups. The length of recruitment was extended due to difficulties recruiting this group. This is perhaps not surprising given that lower socioeconomic groups are generally under-represented in health and medical research [64]. In addition, we did not measure participants’ health literacy skills. While educational attainment is associated with literacy and health literacy, they are not synonymous. Further testing with higher and lower health literacy groups would be an important next step to identify whether the decision aid is suitable and understandable to different health literacy groups.

Thirdly, given the aims of the study were to provide descriptive data on the acceptability of the DA, the results should therefore be considered as preliminary. The small sample size in each education group meant that we were underpowered to perform statistical analyses to detect any statistical differences between the two groups. At this stage, it is not possible to make conclusive statements about the actual effect of the decision aid without a randomised controlled trial with a larger sample, to evaluate the efficacy of the DA compared to standard information. This will provide valuable evidence on the effect of the decision aid on informed decision-making (knowledge, attitudes, uptake, values consistency and deliberation), and enable us to generalise the findings to populations with different education levels.

Finally, in line with previous work [65], there was a very low response rate from GPs creating problems of non-response bias. GPs who responded may have had a stronger interest in prenatal screening and reactions to the DA may have been different among GPs who did not respond. Furthermore, although we asked GPs to invite all eligible pregnant women to participate, GPs may have selected women whom they thought had the necessary health literacy skills to read and understand the DA. We had considerable difficulties recruiting women with lower education levels throughout the study which subsequently delayed recruitment.

### Conclusion

The DA was found to be acceptable, comprehensible, and useful to women from different educational backgrounds and GPs. Women’s knowledge about screening, including NIPT, increased after exposure to the DA. The next steps will be to further test the DA among women with different health literacy skills, and then evaluate the efficacy of the DA on informed decision-making (knowledge, attitudes, values-consistency, deliberation) compared to standard information in a larger sample. It would also be important to identify strategies for how the tool could be more broadly used and implemented by GPs and other health professionals.

### Practice implications

This is one of the few prenatal screening DAs to provide information about NIPT. It has the potential to provide prospective parents with clear and easy-to-read information, and complement existing information presented by health care professionals. A formal evaluation of the efficacy of the tool (compared to standard information) is necessary before it is made available.

Although all participants spoke English, around one-third of participants were bilingual. If translated into different languages, the DA could provide culturally and linguistically diverse (CALD) populations with a tool that is accessible in their own language and help to address the potential communication challenges. Future work could also focus on translating the DA into different languages to address the needs of those from CALD backgrounds.

Future work is also needed to identify and overcome the barriers to implementing DAs, with studies focused on identifying the contextual and facilitative mechanisms that could influence the implementation of the DA [66]. The potential use of social marketing, (the use of commercial marketing strategies to enhance public health and well-being [67], has also been suggested [57]. Future work could be directed towards identifying social marketing strategies (e.g. social media) to support the long-term implementation of DAs. We also note that this DA was developed in the context of the Australian healthcare system and the risk information presented is based on Australian data. The DA would need modification if tested in other countries.

## Additional file

Additional file 1: Knowledge scoring scheme – questions and scoring scheme for the knowledge measure assessing conceptual and numeric knowledge of prenatal screening. (DOCX 20 kb)

## Abbreviations

DA: Decision aid
NIPT: Non-invasive prenatal testing
