# Supplementary material for: Improving women’s knowledge about prenatal screening in the era of non-invasive prenatal testing for Down syndrome – development and acceptability of a low literacy decision aid
Source: BMC Pregnancy Childbirth. 2018 Dec 17;18:499. doi: 10.1186/s12884-018-2135-0 (PMC6296052; doi:10.1186/s12884-018-2135-0)
Supplement: Supplementary file 1 — Knowledge scoring scheme – questions and scoring scheme for the knowledge measure assessing conceptual and numeric knowledge of prenatal screening. (DOCX 20 kb) [file 12884_2018_2135_MOESM1_ESM.docx]

**Additional File 1. Questions and scoring scheme for the knowledge measure**

| **Core knowledge items** | **Conceptual and numeric knowledge questions** | **Actual answer** | **Marking/scoring scheme**^1^ | | **Maximum mark awarded** |
| --- | --- | --- | --- | --- | --- |
| 1. Awareness that prenatal screening is offered as a choice | 1. In Australia, all women are required to have prenatal screening for Down syndrome | False | True = 0 marks  False = 1 mark | | **1**  Conceptual |
| 1. Awareness that prenatal screening is screening for Down syndrome | 1. Prenatal screening in early pregnancy can tell you the chance that your baby has Down syndrome | True | True = 1 mark  False = 0 marks | | **1**  Conceptual |
| 1. Awareness of the low chance of having a baby with Down syndrome | 1. The chance of having a baby with Down syndrome is generally low (less than 1%) | True | True = 1 mark  False = 0 marks | | **1**  Conceptual |
| 1. Awareness that children with Down syndrome have learning difficulties | 1. Children with Down syndrome have learning difficulties | True | True = 1 mark  False = 0 marks | | **1**  Conceptual |
| 1. Awareness that other chromosome problems can be screened for | 1. Down syndrome is the only chromosome problem that can be screened for in pregnancy | False | True = 0 marks  False = 1 mark | | **1**  Conceptual |
| 1. Awareness of what the combined first trimester screening involves | 1. The combined first trimester screening involves an ultrasound scan to measure the thickness of the baby’s neck and a blood test | True | True = 1 mark  False = 0 marks | | **1**  Conceptual |
| 1. The combined first trimester test is more accurate than the second trimester test | 1. Can you tell me which screening test you think is more accurate in detecting Down syndrome – combined first trimester screening or the second trimester blood test? | Combined first trimester | Combined first trimester = 1 mark  Second trimester blood test = 0 marks | | **1**  Conceptual |
| 1. Sensitivity of the combined first trimester screening test ^2^ | 1. Out of 100 pregnancies with Down syndrome, approximately how many will be found accurately detected by the combined first trimester screening test – ultrasound and blood test? | 90 | 90 = 2 marks  85 – 89 = 1 mark  91 – 93 = 1 mark  Other/ don’t know = 0 marks | | **2**  Numeric |
| 1. Sensitivity of the second trimester screening test ^3^ | 1. Out of 100 pregnancies with Down syndrome, approximately how many will be accurately detected by the second trimester screening – the blood test? | 75 | 75 = 2 marks  64 – 74 = 1 mark^2^  76 – 78 = 1 mark^2^  Other/ don’t know = 0 marks | | **2**  Numeric |
| 1. Awareness of what the NIPT involves | 1. Non-invasive prenatal testing (NIPT) involves a pregnant woman having a blood test | True | True = 1 mark  False = 0 marks | | **1**  Conceptual + NIPT |
| 1. Awareness that NIPT is not a diagnostic test | 1. Non-invasive prenatal testing (NIPT) will confirm that your baby has Down syndrome | False | True = 0 marks  False = 1 mark | | **1**  Conceptual +NIPT |
| 1. NIPT is more accurate than the first and second trimester screening tests | 1. Non-invasive prenatal testing (NIPT) is more accurate in accurately detecting pregnancies with Down syndrome than the combined first trimester screening test and second trimester screening test | True | True = 1 mark  False = 0 marks | | **1**  Conceptual +NIPT |
| 1. Sensitivity of the NIPT ^4^ | 1. Out of 100 pregnancies with Down syndrome, approximately how many will be accurately detected by Non-invasive prenatal testing (NIPT)? | 99 | 99 – 99.6 = 2 marks  95 – 98.9 = 1 mark  99.6 – 99.9 = 1 mark  Other/ don’t know = 0 marks | | **2**  Numeric + NIPT |
| 1. Awareness that screening accuracy is not dependent on maternal age | 1. Overall, screening tests are more accurate in detecting pregnancies with Down syndrome in women who are younger | False | True = 0 marks  False = 1 mark | | **1**  Conceptual |
| 1. Awareness that a low risk result does not mean no chance of having a baby with Down syndrome | 1. If a screening test result tells you that you have a ‘low’ risk of having a baby with Down syndrome, there is still a chance that the baby has Down syndrome | True | True = 1 mark  False = 0 marks | | **1**  Conceptual |
| 1. Awareness that a high risk result does not mean the baby does have Down syndrome | 1. If a screening test result tells you that you have a higher chance of having a baby with Down syndrome, it means that the baby has Down syndrome | False | True = 0 marks  False = 1 mark | | **1**  Conceptual |
| 1. Awareness that the diagnostic test confirms if the baby has/does not have Down syndrome | 1. Diagnostic tests such as chorionic villus sampling and amniocenteses confirm/provide reassurance that the baby does or does not have Down syndrome | True | True = 1 mark  False = 0 marks | | **1**  Conceptual |
| 1. Awareness that diagnostic tests have a chance of miscarriage | 1. Diagnostic test such as chorionic villus sampling and amniocentesis have a chance of miscarriage | True | True = 1 mark  False = 0 marks | | **1**  Conceptual |
| 1. Awareness that termination is an option if the baby has Down syndrome | 1. Women have the option of ending their pregnancy if the results of diagnostic tests such as chorionic villus sampling and amniocentesis confirm that the baby has Down syndrome | True | True = 1 mark  False = 0 marks | | **1**  Conceptual |
|  | Maximum conceptual knowledge score  Maximum numerical knowledge score  **Maximum total knowledge Score** | | | 16  6  **22** | |
| ^1^ A response of ‘Don’t know’ was interpreted as 0 marks.  ^2^ Sensitivity of the combined first trimester screening test ranges from 85 – 93%  ^3^ Sensitivity of the second trimester screening test ranges from 64 – 78%  (Figures regarding the sensitivity of combined first trimester screening and second trimester screening were drawn from the report produced by HGSA/RANZCOG Joint Committee on Prenatal Diagnosis and Screening. Prenatal screening and diagnosis of chromosomal and genetic conditions in the fetus in pregnancy. https://www.ranzcog.edu.au  ^4^ Sensitivity of the NIPT ranges from 99.0 – 99.6 (Hyett J, 2014. Non-invasive prenatal testing for Down syndrome, Aust Prescr 2014;37:51-51). | | | |  | |
